# A nomogram for predicting emergence agitation after laparoscopic high ligation of the hernia sac in children

**DOI:** 10.3389/fped.2026.1847049

**Published:** 2026-08-05

**Authors:** Chun Liang, Xifeng Zhang, Lian Zhao

**Affiliations:** 1 Department of Anesthesiology, Jinhua Maternal & Child Health Care Hospital (Jinhua Women’s and Children’s Hospital), Jinhua, Zhejiang, China; 2 Department of Anesthesiology, Children’s Hospital of Nanjing Medical University, Nanjing, Jiangsu, China; 3 Department of Anesthesiology, Huai'an Hospital of Huai'an City, (Huai'an Clinical Medical College of Jiangsu University), Huai'an, Jiangsu, China

**Keywords:** clinical prediction model, emergence agitation, nomogram, pediatric anesthesia, preoperative anxiety

## Abstract

**Background:**

Emergence agitation (EA) is a frequent recovery complication in children and may compromise postoperative safety. This study aimed to develop and internally validate an exploratory model for predicting Pediatric Anesthesia Emergence Delirium (PAED)-defined EA after laparoscopic high ligation of the hernia sac.

**Methods:**

This single-center retrospective prediction-model study screened 342 consecutive procedures performed from January 2022 to December 2024. Complete-case analysis included 287 children. The prediction time was immediately before planned tracheal extubation and before the first PAED assessment. After review of the original data-driven selection results, a *post hoc* four-predictor logistic model was constructed using age, baseline preoperative anxiety, sedative premedication, and intraoperative regional block. Conditional internal validation was performed using 1,000 bootstrap resamples.

**Results:**

PAED-defined EA occurred in 93 children (32.4%). Increasing age was associated with lower odds of EA, whereas baseline preoperative anxiety was associated with higher odds; sedative premedication and intraoperative regional block were associated with lower odds. The model showed an apparent area under the receiver operating characteristic curve of 0.768 (95% confidence interval, 0.710–0.826) and an optimism-corrected value of 0.755. The optimism-corrected calibration slope and intercept were 0.943 and −0.028, respectively, and the corrected Brier score was 0.185.

**Conclusions:**

The *post hoc* four-predictor nomogram showed moderate discrimination and acceptable internal calibration for estimating PAED-defined EA risk. Independent external validation and prospective clinical impact assessment are required before implementation.

## 1 Introduction

Laparoscopic high ligation of the hernia sac is a commonly used minimally invasive procedure for pediatric inguinal hernia and is associated with small incisions, rapid recovery, and a low recurrence rate (1). With the increasing use of pediatric day surgery and short-stay pathways, the quality of early postoperative recovery has become an important outcome of anesthetic management. Emergence agitation (EA) is a common behavioral disturbance during recovery from general anesthesia in children and is characterized by crying, motor restlessness, disorientation, inconsolability, and purposeless movements. A recent meta-analysis showed that its incidence varies substantially according to age, surgical procedure, anesthetic technique, and assessment criteria (2). EA may increase safety risks during recovery, including inadvertent removal of intravenous access, falls, wound traction, prolonged stay in the post-anesthesia care unit (PACU), and increased parental anxiety (3). Factors available before EA occurs, including age, preoperative anxiety, and exposure to inhalational anesthetics, have been associated with a higher risk of EA in children (4). Postoperative pain and EA frequently occur concurrently during early recovery and may trigger one another and jointly influence behavioral presentation; therefore, pain scores during emergence are more appropriately treated as recovery-phase safety-monitoring information than as predictors available before the event (5).

Although laparoscopic high ligation of the hernia sac is generally brief and causes limited tissue trauma, the procedure is performed predominantly in young children, in whom perioperative separation anxiety and stimulation from an unfamiliar environment are common. Inhalational anesthetics with low blood-gas partition coefficients, such as sevoflurane, allow rapid emergence but may also be accompanied by behavioral fluctuations during recovery.

Existing prediction studies of pediatric EA remain limited with respect to candidate-variable determination, data-driven selection procedures, calibration reporting, and external validation (6–10). Clinical prediction-model studies should clearly specify when candidate variables are measured, how they are coded, the rules used for selection, and which analytical steps are included in internal validation (7–10). In this retrospective analysis of children undergoing laparoscopic high ligation of the hernia sac, the prediction time was defined as immediately before planned tracheal extubation and before the first Pediatric Anesthesia Emergence Delirium (PAED) scale assessment. We report the original pathway of univariable screening and backward likelihood-ratio selection and, after reviewing the original analytical results, developed a *post hoc* four-predictor nomogram based on availability at the prediction time, nonredundancy across variable domains, completeness of documentation, and parsimony of presentation. Conditional internal validation of the fixed four-variable model and nested bootstrap validation of the original algorithmic selection pathway were conducted separately. The objective was to develop an exploratory candidate model for independent external validation.

## 2 Materials and methods

### 2.1 Study design and ethics

This was a single-center retrospective prediction-model study comprising *post hoc* exploratory model development and internal validation. The prediction time was defined as immediately before planned tracheal extubation and before the first PAED assessment. The study protocol was approved by the Medical Ethics Committee of Jinhua Maternal and Child Health Care Hospital (Jinhua Women and Children's Hospital) (approval no. QT-023 [2026]) and was conducted in accordance with applicable ethical principles, local regulations, and institutional requirements (11). Because the study involved only retrospective use of existing clinical records and introduced no additional diagnostic or therapeutic intervention, the ethics committee waived the requirement for written informed consent from the children's legal guardians.

### 2.2 Study population

**Figure 1:**
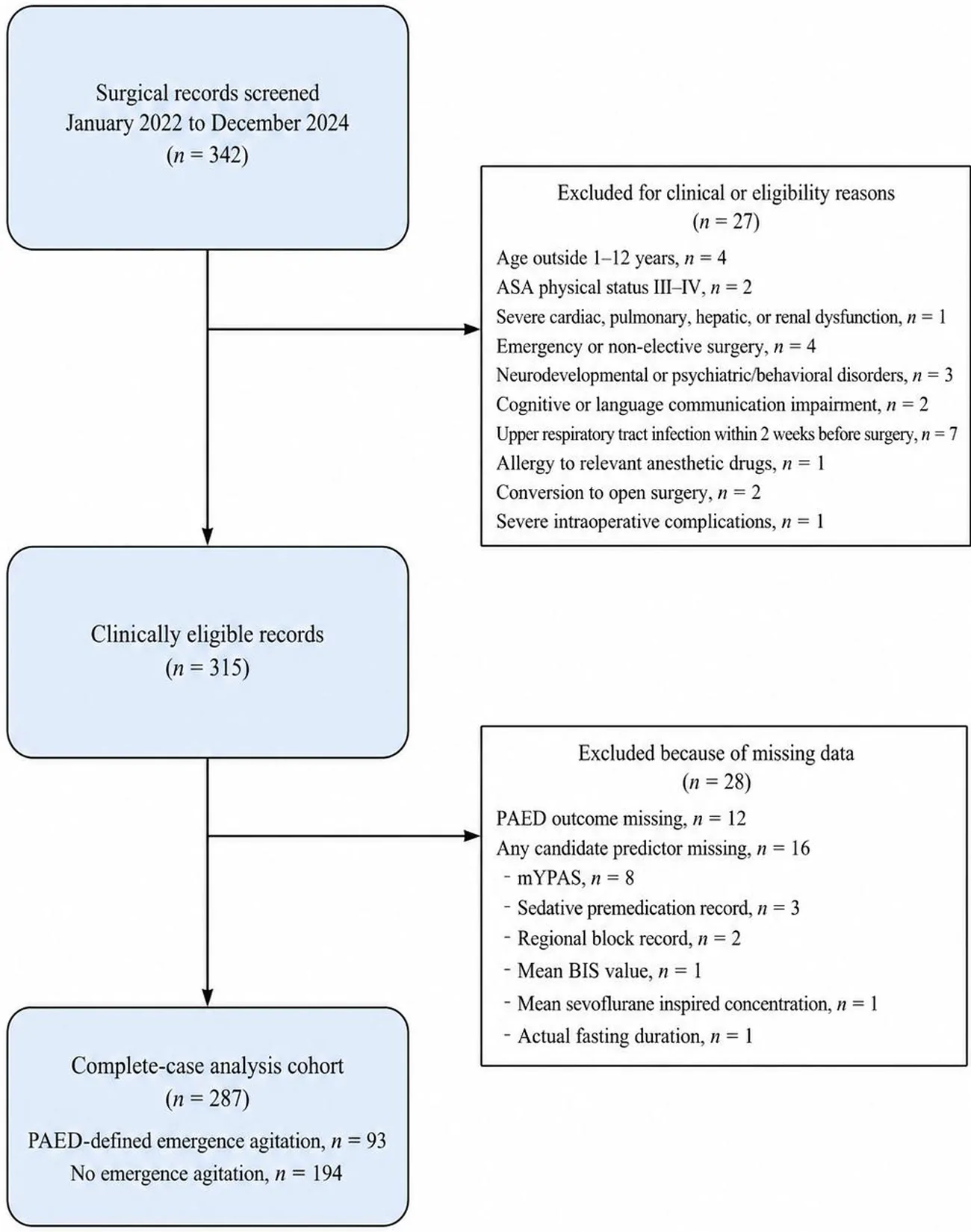
Flow of participant screening, clinical/eligibility exclusions, missing analysis variables, and complete-case analysis. Clinical exclusions and missing fields were counted at the patient level using the first applicable reason to ensure mutually exclusive counts. ASA, American Society of Anesthesiologists; PAED, Pediatric Anesthesia Emergence Delirium; mYPAS, modified Yale Preoperative Anxiety Scale; BIS, bispectral index; EA, emergence agitation.

Records of 342 consecutive procedures performed at our hospital from January 2022 through December 2024 were screened. The clinical inclusion criteria were: (1) age 1–12 years; (2) American Society of Anesthesiologists (ASA) physical status I–II; and (3) elective laparoscopic high ligation of the hernia sac. The clinical exclusion criteria were: (1) severe cardiac, pulmonary, hepatic, or renal dysfunction; (2) neurodevelopmental abnormalities or psychiatric/behavioral disorders; (3) cognitive or language-communication impairment that affected scale assessment; (4) upper respiratory tract infection within 2 weeks before surgery; (5) allergy to relevant anesthetic drugs; (6) emergency or non-elective surgery; (7) conversion to open surgery; (8) severe intraoperative complications; and (9) ASA physical status III–IV or age outside the specified range. Each patient was assigned to the first applicable reason so that the counts were mutually exclusive. After assessment of clinical eligibility, 315 records underwent data-completeness review, and 287 complete cases were included in the primary analysis (Figure 1).

### 2.3 Preoperative assessment, sedative premedication, anesthesia, and surgery

All children complied with preoperative fasting and clear-fluid restriction requirements. In the preoperative holding area, an anesthesia nurse or anesthesiologist who had received standardized training in administration of the scale assessed each child using the modified Yale Preoperative Anxiety Scale (mYPAS). The assessment was performed 30–60 min before anticipated anesthesia induction and before any oral, intranasal, or intravenous sedative premedication. This score was used as the baseline measure of preoperative anxiety.

Sedative premedication was defined as complete documentation in the medical record of one of the following mutually exclusive regimens before anesthesia induction: (1) intranasal dexmedetomidine 1.0 μg/kg, maximum 40 μg, administered 30–45 min before induction; (2) oral midazolam 0.5 mg/kg, maximum 15 mg, administered 20–30 min before induction; or (3) intravenous midazolam 0.05 mg/kg, maximum 2 mg, administered 5–10 min before induction after establishment of patent peripheral intravenous access. Documented indications for the intranasal or oral regimens included a baseline mYPAS score >30, marked separation anxiety, and anticipated difficulty with transfer, venipuncture, or cooperation during anesthesia induction. Intravenous midazolam was used in children who already had patent intravenous access and continued to show marked anxiety or difficulty cooperating during induction. The specific regimen was selected after consideration of age, fasting status, airway status, the attending anesthesiologist's judgment, and parental preference. The drug, route, actual dose, administration time, and documented indication were abstracted from the preanesthetic assessment, medication orders, and anesthesia record. Each child received only one regimen; in the primary model, receipt of any regimen was coded as 1 and no regimen as 0.

Peripheral intravenous access was established in the preoperative holding area or after entry into the operating room but before anesthesia induction; intravenous midazolam was administered only after patency had been confirmed. Standard intraoperative monitoring included electrocardiography, pulse oximetry, noninvasive blood pressure, and end-tidal carbon dioxide. Anesthesia was induced with atropine 0.01 mg/kg, propofol 2–3 mg/kg, sufentanil 0.2–0.3 μg/kg, and cisatracurium 0.1–0.15 mg/kg. After tracheal intubation, mechanical ventilation was used to maintain end-tidal carbon dioxide at 35–45 mmHg. Anesthesia was maintained with 1.5%–3.0% sevoflurane, with intermittent cisatracurium supplementation, and the bispectral index (BIS) was maintained at 40–60. Surgery was performed using a standard three-port technique with a pneumoperitoneum pressure of 6–10 mmHg. Sufentanil 0.1 μg/kg was administered intravenously 10 min before the end of surgery. Some children received an ultrasound-guided transversus abdominis plane block or rectus sheath block with 0.25% ropivacaine 0.5 mL/kg; the specific block technique and dose were abstracted from the intraoperative anesthesia record (12, 13).

### 2.4 Data collection, prediction time, and outcome definition

General variables included sex, age, weight, height, and body mass index (BMI). Preoperative variables included ASA physical status, hernia type and laterality, history of incarcerated hernia, previous surgery and anesthesia, baseline preoperative anxiety, sedative premedication, actual fasting duration, clear-fluid fasting duration, parental presence, and child temperament. Baseline preoperative anxiety was assessed with the mYPAS before any sedative premedication; an mYPAS score >30 defined baseline preoperative anxiety (14, 15). Sedative premedication was defined as receipt before induction of intranasal dexmedetomidine, oral midazolam, or intravenous midazolam, and the drug, route, actual dose, administration time, and documented indication were recorded.

The outcome was assessed using the Pediatric Anesthesia Emergence Delirium (PAED) scale. PAED scores were recorded immediately after tracheal extubation and at 5, 10, 15, 20, 25, and 30 min after admission to the PACU; the highest score recorded within 30 min was used. A maximum score ≥10 defined PAED-defined EA (16, 17). Emergence-phase pain was recorded as PACU safety-monitoring information and was assessed according to age using the Face, Legs, Activity, Cry, and Consolability scale, the Wong-Baker FACES Pain Rating Scale, or a visual analog scale (18). Child temperament was reported by parents in the preoperative clinic using an age-appropriate questionnaire from the Carey series according to age on the day of surgery: the Toddler Temperament Scale for children aged 1 to <3 years, the Behavioral Style Questionnaire for those aged 3–7 years, and the Middle Childhood Temperament Questionnaire for those aged 8–12 years (19–21). Each questionnaire was scored according to its specific rules and classified as easy, difficult, slow-to-warm-up, intermediate-easy, or intermediate-difficult.

Intraoperative variables included surgical duration, anesthesia duration, total sevoflurane consumption and mean inspired concentration, total sufentanil dose, cisatracurium dose, additional intraoperative opioid use, intraoperative fluid volume, blood loss, duration and pressure of pneumoperitoneum, hemodynamic fluctuations, intraoperative adverse events, minimum and mean BIS values, and regional block technique. The prediction time was defined as immediately before planned extubation and before the first PAED assessment. At that time, age, baseline preoperative anxiety, sedative premedication, intraoperative regional block, and all intraoperative candidate variables had been recorded, whereas the PAED outcome had not yet been assessed. The fixed four-variable model estimated the conditional risk of PAED-defined EA immediately before tracheal extubation, after completion of the specified preoperative and intraoperative management.

### 2.5 Statistical analysis

Statistical analyses were performed using Statistical Package for the Social Sciences (SPSS; IBM Corp., Armonk, NY, USA), version 26.0, and R software, version 4.2.0. Normality of continuous variables was assessed using the Shapiro–Wilk test. Normally distributed variables were summarized as mean ± standard deviation and compared using Student's *t* test or Welch-corrected *t* test, as appropriate according to homogeneity of variance. Non-normally distributed variables were summarized as median (interquartile range) and compared using the Mann–Whitney *U* test. Categorical variables were summarized as frequency (percentage) and compared using the Pearson *χ^2^* test or Fisher's exact test; no continuity correction was applied to 2 × 2 contingency tables. All tests were two-sided, and *P* < 0.05 was considered statistically significant.

Missing-data review covered the PAED outcome and all 16 candidate predictors. Missing PAED outcomes were identified first; thereafter, each record was assigned to the first missing field in a predefined sequence—mYPAS, sedative premedication record, regional block, mean BIS, mean inspired sevoflurane concentration, actual fasting duration, and the remaining candidate fields—to produce mutually exclusive patient-level counts. Of the 315 records meeting the clinical inclusion and exclusion criteria, 28 (8.9%) lacked data required for analysis: 12 had missing PAED outcomes and 16 had at least one missing candidate predictor. The primary analysis included 287 complete cases. Neither single nor multiple imputation was performed, and records with missing data were excluded from model estimation.

The candidate-predictor set was limited to 16 variables available by the prediction time and reproducibly extractable from routine medical records. Continuous variables entered the model in their original units: age (years), BMI (kg/m^2^), actual fasting duration (h), clear-fluid fasting duration (h), mean inspired sevoflurane concentration (%), total sufentanil dose (*μ*g), and mean intraoperative BIS. Binary variables were coded 1/0: male sex, ASA physical status II, bilateral hernia, baseline preoperative anxiety, sedative premedication, parental presence, at-risk temperament (difficult or slow-to-warm-up vs. other categories), additional intraoperative opioid use, and intraoperative regional block. Weight and height were not entered simultaneously into the candidate model because their information overlapped with age and BMI. Laterality of unilateral hernia, the specific type of regional block, and low-frequency intraoperative adverse events were described only.

The original analysis fitted a separate univariable logistic regression model for each of the 16 candidate variables. A likelihood-ratio test *P* < 0.10 was the criterion for entry into the multivariable model, followed by backward likelihood-ratio selection with removal at *P* > 0.10. The 12 variables meeting the entry criterion were age, baseline preoperative anxiety, sedative premedication, actual fasting duration, clear-fluid fasting duration, parental presence, at-risk temperament, mean inspired sevoflurane concentration, total sufentanil dose, additional intraoperative opioid use, mean intraoperative BIS, and intraoperative regional block. After backward selection, 11 variables were retained; additional intraoperative opioid use was removed. After the original results had been reviewed, age, baseline preoperative anxiety, sedative premedication, and intraoperative regional block were selected *post hoc* to construct a fixed exploratory model based on availability at the prediction time, nonredundancy across variable domains, completeness of documentation, and parsimony of presentation. The four predictors corresponded to four degrees of freedom, and the 93 EA events yielded an apparent events per variable (EPV) of 23.3. This metric describes only the parameter burden of the final fixed model.

EA was coded as 1 and non-EA as 0. Age was entered as a continuous variable in completed years, with each 1-year increment representing one unit; baseline preoperative anxiety, sedative premedication, and intraoperative regional block were each coded as yes = 1 and no = 0. The linear predictor (LP) was LP = 0.660−0.326 × age + 0.971 × baseline preoperative anxiety−0.814 × sedative premedication−0.991 × intraoperative regional block. The individual predicted probability of PAED-defined EA was calculated as *P* (EA) = exp (LP)/[1 + exp (LP)] = 1/[1 + exp (−LP)]. Nomogram points and the risk scale were derived from the same equation.

The fixed four-predictor model underwent conditional internal validation using 1,000 bootstrap resamples. This analysis treated the four-variable specification as fixed. In each bootstrap sample, coefficients for the same four predictors were re-estimated, apparent performance was calculated in the bootstrap sample, and the resulting model was then applied to the original sample to calculate test performance; the difference was used to estimate optimism. Mean optimism was used to correct the original-sample area under the receiver operating characteristic curve (AUC) and Brier score, and the calibration intercept and slope were evaluated within the same framework. The original data-driven selection pathway underwent nested bootstrap internal validation. Each bootstrap sample began with all 16 candidate predictors and repeated univariable likelihood-ratio screening at *P* < 0.10 and backward likelihood-ratio selection with removal at *P* > 0.10. The final model was re-estimated within that resample and applied to the original sample to calculate the test AUC. The number of predictors ultimately retained, the selection frequency of each predictor, and the optimism-corrected AUC for the complete algorithmic pathway were recorded.

Model discrimination was quantified using the AUC and its DeLong 95% confidence interval (CI). The calibration plot simultaneously displayed the ideal relationship, apparent decile calibration, and the bootstrap-corrected relationship; the calibration intercept, calibration slope, and Brier score were also reported. Youden J (sensitivity + specificity−1) was calculated from the receiver operating characteristic coordinates in the development sample. The unrounded predicted-probability cutoff corresponding to the maximum Youden J was 0.348 and was reported as 0.35 to two decimal places. Because no study participant had a predicted probability in the interval 0.348 ≤ predicted probability < 0.350, classification at 0.35 yielded the same 2 × 2 table. A predicted probability ≥0.35 was defined as a positive classification. True positives (TP), false negatives (FN), true negatives (TN), false positives (FP), sensitivity, specificity, positive predictive value, and negative predictive value were reported; 95% CIs for proportions were calculated using the Wilson method.

Exploratory subgroup analyses were stratified by age (<4 vs. ≥4 years), sex, BMI (the 85th percentile in this cohort, 18.22 kg/m^2^), ASA physical status, and hernia type. For each subgroup, predicted probabilities from the fixed four-variable model were used to calculate the AUC, with 95% CIs obtained from 2,000 outcome-stratified bootstrap resamples. The AUC difference between the two levels of each binary subgroup and its two-sided *P* value were estimated from the same 2,000-resample distribution of differences; no adjustment for multiplicity was applied. The sensitivity analysis used Firth penalized-likelihood logistic regression with the same four predictors as the primary model. Odds ratios (ORs) were obtained by exponentiating the penalized coefficient estimates, 95% CIs were derived using profile penalized likelihood, and *P* values were obtained using penalized likelihood-ratio tests.

## 3 Results

### 3.1 Participant flow and incidence of EA

A total of 342 surgical records were screened consecutively. Twenty-seven records were excluded for clinical or eligibility reasons: age outside 1–12 years (*n* = 4), ASA physical status III–IV (*n* = 2), severe organ dysfunction (*n* = 1), emergency or non-elective surgery (*n* = 4), neurodevelopmental or psychiatric/behavioral disorders (*n* = 3), cognitive or language-communication impairment (*n* = 2), upper respiratory tract infection within 2 weeks before surgery (*n* = 7), allergy to relevant anesthetic drugs (*n* = 1), conversion to open surgery (*n* = 2), and severe intraoperative complications (*n* = 1). Among the 315 clinically eligible records, the PAED outcome was missing in 12 and at least one candidate predictor was missing in 16 (mYPAS, *n* = 8; sedative premedication record, *n* = 3; regional block record, *n* = 2; mean BIS, *n* = 1; mean inspired sevoflurane concentration, *n* = 1; and actual fasting duration, *n* = 1). The final analysis included 287 complete cases, of whom 93 had PAED-defined EA and 194 did not; the incidence was 32.4% (Figure 1).

### 3.2 Demographic and preoperative characteristics of the two groups

The groups did not differ significantly in sex, weight, height, BMI, ASA physical status, hernia type, laterality among unilateral hernias, history of incarcerated hernia, or previous surgery/anesthesia (all *P* > 0.05). Children in the EA group were younger, had a higher proportion of baseline preoperative anxiety, a lower proportion of sedative premedication, longer actual fasting and clear-fluid fasting durations, a lower proportion of parental presence, and a different distribution of temperament categories. Overall, 106 children (36.9%) received sedative premedication, and each child received only one regimen: intranasal dexmedetomidine in 63 (59.4%), oral midazolam in 31 (29.2%), and intravenous midazolam in 12 (11.3%). Among the 24 medicated children in the EA group, 12 received intranasal dexmedetomidine, 9 received oral midazolam, and 3 received intravenous midazolam; the corresponding counts among the 82 medicated children in the non-EA group were 51, 22, and 9. The distribution of regimens was reported descriptively only (Table 1).

**Table 1:** Demographic and preoperative characteristics of the two groups.

Variable	Emergence agitation (EA) group, *n* = 93	No EA group, *n* = 194	Statistic	*P* value
Sex: male	71 (76.3)	142 (73.2)	*χ^2^* = 0.326	0.568
Sex: female	22 (23.7)	52 (26.8)		
Age, years, mean ± standard deviation (SD)	3.4 ± 1.6	4.6 ± 2.2	Welch *t* = 5.238	<0.001
Weight, kg, mean ± SD	17.5 ± 5.8	18.7 ± 6.8	Welch *t* = 1.549	0.123
Height, cm, mean ± SD	98.6 ± 14.2	102.3 ± 16.8	Welch *t* = 1.944	0.053
Body mass index (BMI), kg/m^2^, mean ± SD	15.6 ± 2.3	15.9 ± 2.5	Welch *t* = 1.005	0.316
American Society of Anesthesiologists (ASA) physical status I	76 (81.7)	165 (85.1)	*χ^2^* = 0.518	0.472
ASA physical status II	17 (18.3)	29 (14.9)		
Unilateral hernia	59 (63.4)	139 (71.6)	*χ^2^* = 1.980	0.159
Bilateral hernia	34 (36.6)	55 (28.4)		
Left-sided unilateral hernia^a^	24 (40.7)	63 (45.3)	*χ^2^* = 0.363	0.547
Right-sided unilateral hernia^a^	35 (59.3)	76 (54.7)		
History of incarcerated hernia	8 (8.6)	12 (6.2)	*χ^2^* = 0.566	0.452
Previous surgery/anesthesia	11 (11.8)	18 (9.3)	*χ^2^* = 0.450	0.502
Baseline preoperative anxiety (modified Yale Preoperative Anxiety Scale [mYPAS] > 30)	58 (62.4)	67 (34.5)	*χ^2^* = 19.804	<0.001
Sedative premedication (any regimen)	24 (25.8)	82 (42.3)	*χ^2^* = 7.314	0.007
Intranasal dexmedetomidine^b^	12 (50.0)	51 (62.2)	Descriptive	–
Oral midazolam^b^	9 (37.5)	22 (26.8)	Descriptive	–
Intravenous midazolam^b^	3 (12.5)	9 (11.0)	Descriptive	–
Actual fasting duration, h, mean ± SD	9.8 ± 2.4	9.1 ± 2.1	Welch *t* = 2.406	0.017
Clear-fluid fasting duration, h, mean ± SD	4.6 ± 1.8	4.1 ± 1.5	Welch *t* = 2.320	0.022
Parental presence	51 (54.8)	137 (70.6)	*χ^2^* = 6.928	0.008
Temperament: easy	18 (19.4)	68 (35.1)	*χ^2^* = 15.021	0.005
Temperament: difficult	29 (31.2)	32 (16.5)		
Temperament: slow-to-warm-up	21 (22.6)	28 (14.4)		
Temperament: intermediate-easy	14 (15.1)	38 (19.6)		
Temperament: intermediate-difficult	11 (11.8)	28 (14.4)		

Data are presented as mean ± standard deviation or *n* (%).

Temperament categories were assessed using age-appropriate questionnaires from the Carey series covering ages 1–12 years [19–21]. Welch *t* denotes the Welch-corrected independent-samples *t* statistic; Pearson *χ^2^* denotes the Pearson chi-square statistic; Fisher denotes Fisher's exact test. All *P* values are two-sided.

*n*, number of children; EA, emergence agitation; mYPAS, modified Yale Preoperative Anxiety Scale; BMI, body mass index; ASA, American Society of Anesthesiologists; SD, standard deviation.

a Laterality was compared only among children with unilateral hernia.

b Percentages for the three drug regimens use as denominators the 24 medicated children in the EA group and the 82 medicated children in the non-EA group and are reported descriptively only; the mYPAS was completed before medication, and the three regimens were mutually exclusive.

### 3.3 Intraoperative characteristics of the two groups

The groups did not differ significantly in surgical duration, anesthesia duration, total sevoflurane consumption, cisatracurium dose, intraoperative fluid volume, blood loss, pneumoperitoneum duration or pressure, maximum or minimum intraoperative heart rate, maximum or minimum intraoperative systolic blood pressure, intraoperative hypoxemia, hypotension, bradycardia, or type of regional block (all *P* > 0.05). The EA group had a higher mean inspired sevoflurane concentration and lower total sufentanil dose, lower frequency of additional intraoperative opioid use, lower minimum and mean BIS values, and a lower frequency of intraoperative regional block. These differences were statistically significant (mean inspired sevoflurane concentration, minimum BIS, mean BIS, and intraoperative regional block, all *P* < 0.001; total sufentanil dose, *P* = 0.005; and additional intraoperative opioid use, *P* = 0.021) (Table 2).

**Table 2:** Intraoperative characteristics of the two groups.

Variable	Emergence agitation (EA) group, *n* = 93	No EA group, *n* = 194	Statistic	*P* value
Surgical duration, min, mean ± standard deviation (SD)	23.5 ± 8.2	24.8 ± 9.1	Welch *t* = 1.212	0.227
Anesthesia duration, min, mean ± SD	42.6 ± 10.5	44.2 ± 11.8	Welch *t* = 1.160	0.248
Total sevoflurane consumption, mL	18.4 ± 5.6	19.2 ± 6.1	Welch *t* = 1.100	0.273
Mean inspired sevoflurane concentration, %	2.4 ± 0.4	2.2 ± 0.5	Welch *t* = 3.646	<0.001
Total sufentanil dose, μg	5.8 ± 1.9	6.5 ± 2.1	Welch *t* = 2.822	0.005
Cisatracurium dose, mg	2.1 ± 0.7	2.2 ± 0.8	Welch *t* = 1.080	0.281
Additional intraoperative opioid	12 (12.9)	48 (24.7)	*χ^2^* = 5.329	0.021
Intraoperative fluid volume, mL	86.5 ± 28.4	89.2 ± 31.6	Welch *t* = 0.726	0.469
Blood loss, mL, median [first quartile (Q1), third quartile (Q3)]	3 (2, 5)	4 (2, 6)	*Z* = 1.124	0.261
Pneumoperitoneum duration, min	18.2 ± 6.8	19.1 ± 7.4	Welch *t* = 1.019	0.309
Pneumoperitoneum pressure, mmHg	7.8 ± 1.2	7.6 ± 1.1	Welch *t* = 1.357	0.177
Maximum intraoperative heart rate, beats/min	128.5 ± 18.6	126.2 ± 17.8	Welch *t* = 0.994	0.322
Minimum intraoperative heart rate, beats/min	92.4 ± 14.2	94.1 ± 13.8	Welch *t* = 0.958	0.339
Maximum intraoperative systolic blood pressure, mmHg	108.6 ± 12.4	106.8 ± 11.9	Welch *t* = 1.166	0.245
Minimum intraoperative systolic blood pressure, mmHg	78.4 ± 9.6	79.8 ± 10.2	Welch *t* = 1.133	0.259
Minimum intraoperative bispectral index (BIS)	38.4 ± 4.8	41.6 ± 5.2	Welch *t* = 5.143	<0.001
Mean intraoperative BIS	44.2 ± 5.1	46.8 ± 5.6	Welch *t* = 3.914	<0.001
Intraoperative hypoxemia	3 (3.2)	5 (2.6)	Fisher	0.717
Intraoperative hypotension	4 (4.3)	7 (3.6)	Fisher	0.752
Intraoperative bradycardia	2 (2.2)	6 (3.1)	Fisher	1.000
Intraoperative regional block	19 (20.4)	78 (40.2)	*χ^2^* = 10.988	<0.001
Transversus abdominis plane (TAP) block^a^	11 (57.9)	42 (53.8)	*χ^2^* = 0.101	0.751
Rectus sheath block^a^	8 (42.1)	36 (46.2)		

Except for blood loss, continuous variables are presented as mean ± standard deviation; blood loss is presented as median (Q1, Q3), and categorical variables as *n* (%).

*n*, number of children; EA, emergence agitation; SD, standard deviation; BIS, bispectral index; TAP, transversus abdominis plane; Q1, first quartile; Q3, third quartile.

a The type of regional block was compared only among children who received a regional block. Blood loss was compared using the Mann–Whitney *U* test, and *Z* is its standardized statistic. Low-frequency adverse events were compared using Fisher's exact test; other continuous variables were compared using Welch-corrected independent-samples *t* tests, and categorical variables using Pearson chi-square tests. All *P* values are two-sided.

### 3.4 *Post hoc* four-predictor exploratory logistic regression model

Using immediately before planned tracheal extubation and before the first PAED assessment as the prediction time, a *post hoc* four-predictor model comprising age, baseline preoperative anxiety, sedative premedication, and intraoperative regional block was constructed after the original screening and backward-selection results had been reviewed. The 93 PAED-defined EA events and four degrees of freedom yielded an apparent EPV of 23.3. Increasing age was associated with lower odds of EA, baseline preoperative anxiety was associated with higher odds, and sedative premedication and intraoperative regional block were associated with lower odds. All four variables remained statistically associated with the outcome in the exploratory multivariable model. The model intercept was 0.660 [standard error (SE) = 0.396, Wald *χ^2^* = 2.778, *P* = 0.096]. The complete equation was LP = 0.660−0.326 × age + 0.971 × baseline preoperative anxiety−0.814 × sedative premedication−0.991 × intraoperative regional block, and the individual predicted probability was *P* (EA) = 1/[1 + exp (−LP)]. For example, for a 3-year-old child with baseline preoperative anxiety who received neither sedative premedication nor an intraoperative regional block, LP = 0.653, corresponding to a predicted probability of 65.8% (Table 3).

**Table 3:** Four-predictor logistic regression model for emergence agitation defined by the pediatric anesthesia emergence delirium scale.

Variable	Coding	Regression coefficient (β)	Standard error (SE)	Wald *χ^2^*	*P* value	Odds ratio (OR)	95% confidence interval (CI)
Intercept	–	0.660	0.396	2.778	0.096	–	–
Age (per 1-year increase)	Continuous; completed years	−0.326	0.077	17.925	<0.001	0.722	0.621–0.839
Baseline preoperative anxiety	Yes = 1; No = 0	0.971	0.280	12.026	<0.001	2.641	1.525–4.571
Sedative premedication	Yes = 1; No = 0	−0.814	0.304	7.170	0.007	0.443	0.244–0.804
Intraoperative regional block	Yes = 1; No = 0	−0.991	0.317	9.773	0.002	0.371	0.199–0.691

The outcome was coded as EA = 1 and non-EA = 0; binary predictors were coded as yes = 1 and no = 0, and age was entered continuously in completed years. OR = exp (β), and 95% CI = exp[β ± 1.96 × SE]. *P* values are from two-sided Wald tests. The intercept is used to calculate absolute predicted probability; the variable set was determined *post hoc* after the original analytical results had been reviewed.

EA, emergence agitation; β, logistic regression coefficient; SE, standard error; Wald *χ^2^*, Wald chi-square statistic; OR, odds ratio; CI, confidence interval.

### 3.5 Nomogram and model performance

**Figure 2:**
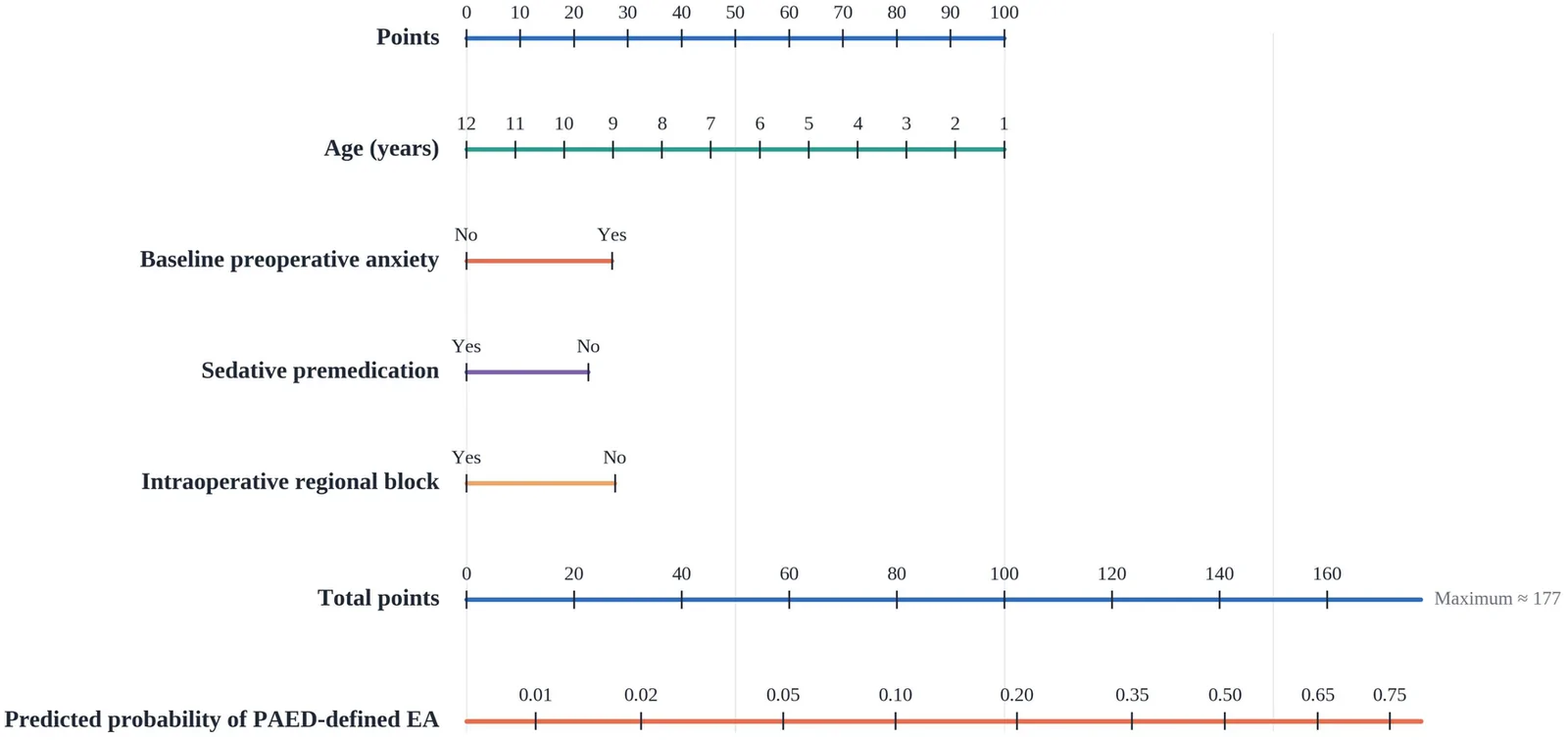
Nomogram for predicting pediatric anesthesia emergence delirium (PAED)-defined emergence agitation (EA) after laparoscopic high ligation of the hernia sac in children. The nomogram corresponds to the complete logistic equation; age is entered in completed years, binary variables are coded as yes = 1 and no = 0, and total points are converted to predicted probability using the same equation.

**Figure 3:**
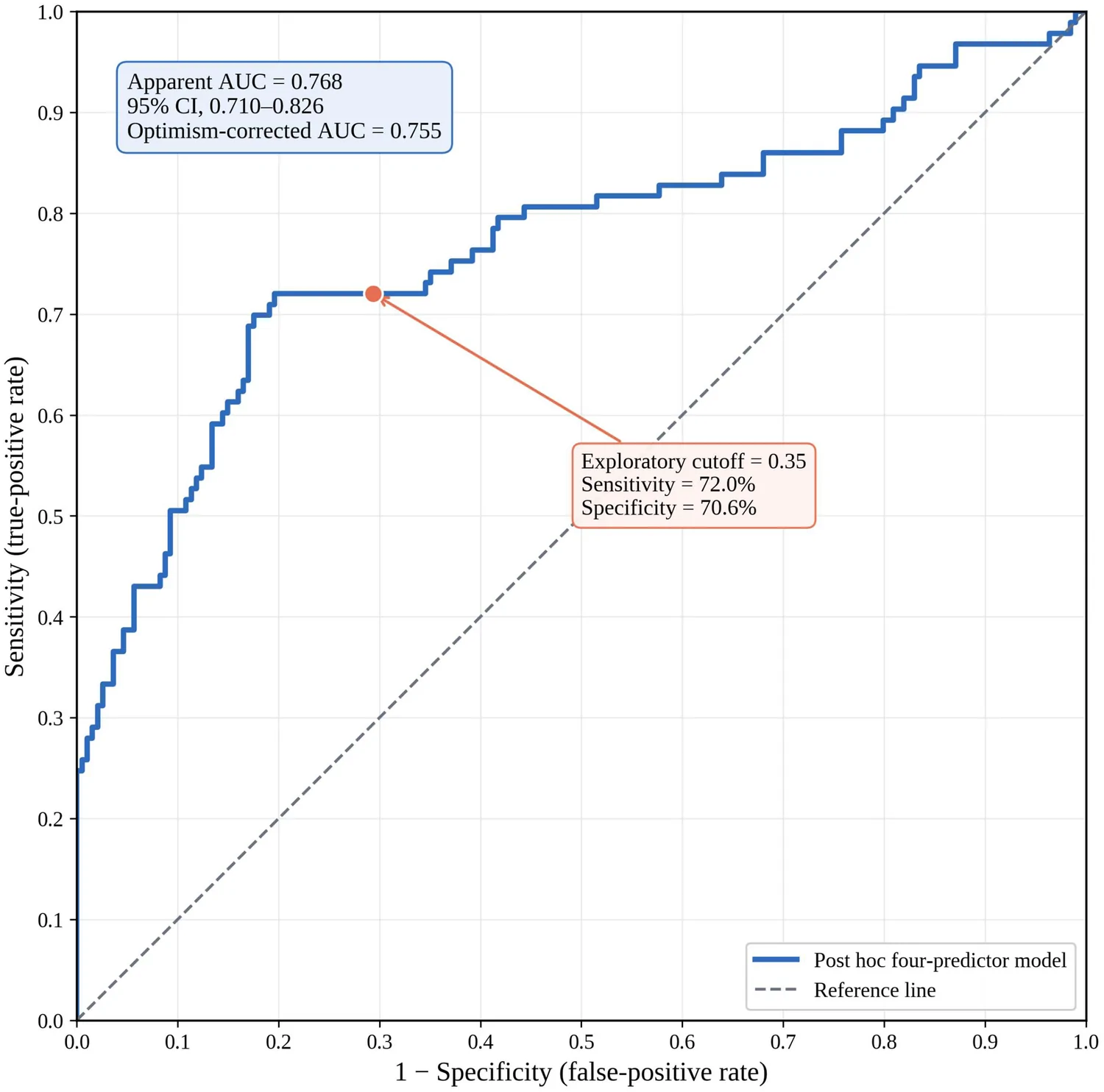
Receiver operating characteristic curve for the *post hoc* four-predictor model. The apparent area under the receiver operating characteristic curve (AUC) was 0.768 (DeLong 95% confidence interval [CI] 0.710–0.826), and the fixed-model optimism-corrected AUC was 0.755. The marker identifies the exploratory development-sample classification cutoff of 0.35, reported to two decimal places, with a sensitivity of 72.0% and a specificity of 70.6%.

**Figure 4:**
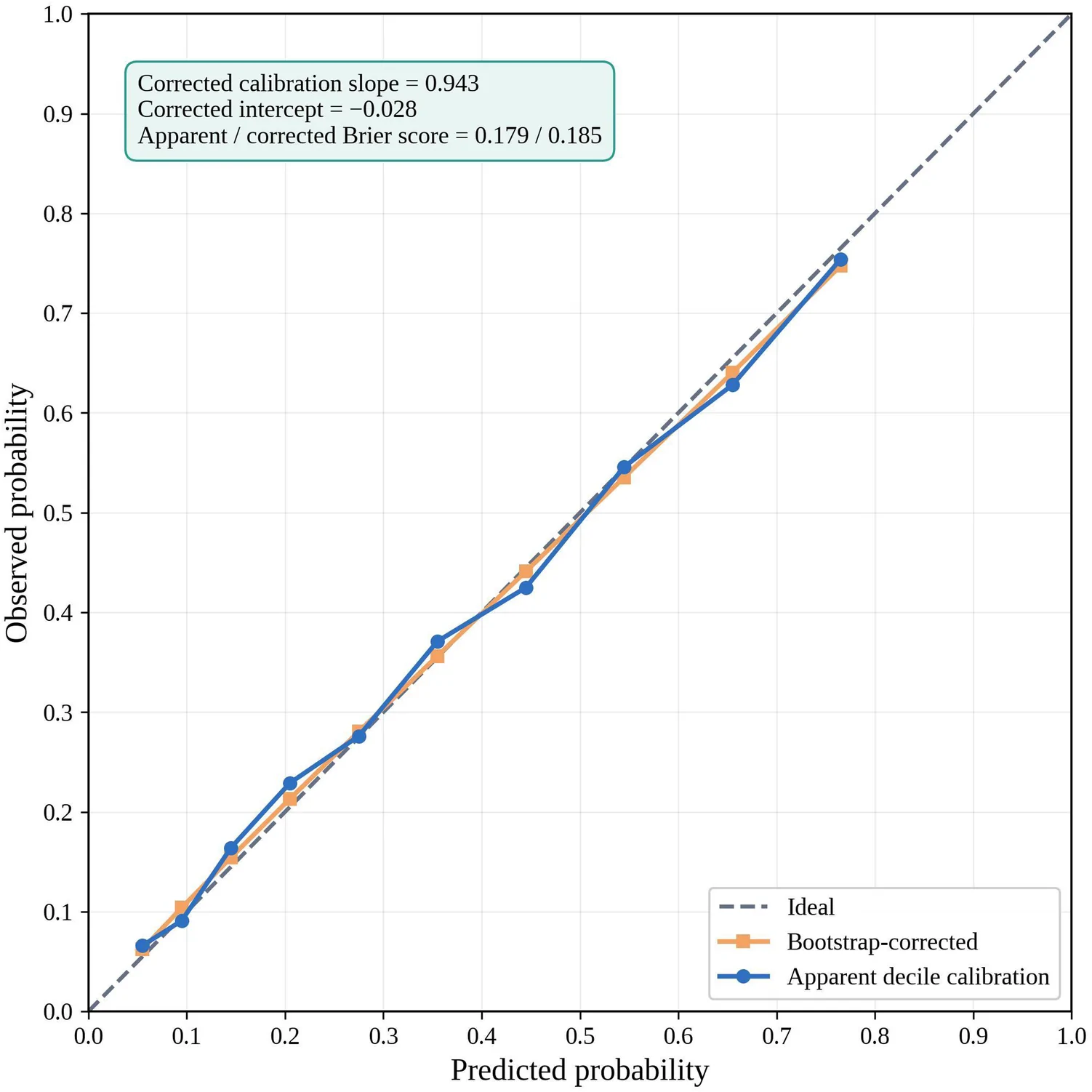
Calibration plot for the *post hoc* four-predictor model. The ideal line, apparent decile calibration, and bootstrap-corrected calibration are shown; the optimism-corrected slope was 0.943, the optimism-corrected intercept was −0.028, and the apparent and optimism-corrected Brier scores were 0.179 and 0.185, respectively.

The apparent AUC of the fixed four-predictor model was 0.768 (DeLong 95% CI 0.710–0.826), and the optimism-corrected AUC based on 1,000 bootstrap resamples was 0.755. The optimism-corrected calibration slope was 0.943 and the calibration intercept was −0.028; the apparent and optimism-corrected Brier scores were 0.179 and 0.185, respectively. The unrounded probability cutoff corresponding to the maximum Youden J in the development sample was 0.348 and was reported as 0.35 to two decimal places. There were 67 true positives, 26 false negatives, 137 true negatives, and 57 false positives. Sensitivity was 72.0% (95% CI 62.2%–80.1%), specificity was 70.6% (95% CI 63.9%–76.6%), positive predictive value was 54.0% (95% CI 45.3%–62.6%), and negative predictive value was 84.0% (95% CI 77.7%–88.9%) (Table 4; Figures 2–4).

**Table 4:** Model performance and conditional internal-validation results.

Metric	Result
Emergence agitation events/total sample size	93/287 (32.4%)
Degrees of freedom in the final exploratory model	4
Apparent events per variable (EPV)	23.3
Apparent area under the receiver operating characteristic curve (AUC)	0.768 (DeLong 95% confidence interval [CI] 0.710–0.826)
Fixed-model bootstrap optimism-corrected AUC	0.755
Optimism-corrected calibration slope	0.943
Optimism-corrected calibration intercept	−0.028
Apparent Brier score	0.179
Optimism-corrected Brier score	0.185
Exploratory data-derived classification cutoff	Unrounded 0.348; reported as 0.35 to two decimal places (predicted probability ≥0.35 classified as positive)
True positive/false negative/true negative/false positive (TP/FN/TN/FP)	67/26/137/57
Sensitivity	72.0% (67/93; 95% CI 62.2%–80.1%)
Specificity	70.6% (137/194; 95% CI 63.9%–76.6%)
Positive predictive value	54.0% (67/124; 95% CI 45.3%–62.6%)
Negative predictive value	84.0% (137/163; 95% CI 77.7%–88.9%)

The unrounded predicted probability corresponding to the maximum Youden J (sensitivity + specificity−1) was 0.348 and was reported as the exploratory data-derived classification cutoff of 0.35 to two decimal places; predicted probability ≥0.35 was defined as a positive classification. The 95% CI for the AUC was calculated using the DeLong method, and 95% CIs for classification metrics were calculated using the Wilson method. A calibration intercept of 0 and slope of 1 indicate ideal calibration. The Brier score ranges from 0 to 1, with lower values indicating less overall prediction error. Calibration metrics and the corrected Brier score were derived from conditional internal validation with 1,000 bootstrap resamples.

EA, emergence agitation; EPV, events per variable; AUC, area under the receiver operating characteristic curve; CI, confidence interval; TP, true positive; FN, false negative; TN, true negative; FP, false positive.

### 3.6 Original selection pathway, subgroup performance, and sensitivity analysis

In univariable logistic regression, 12 variables met the likelihood-ratio *P* < 0.10 criterion: age, baseline preoperative anxiety, sedative premedication, actual fasting duration, clear-fluid fasting duration, parental presence, at-risk temperament, mean inspired sevoflurane concentration, total sufentanil dose, additional intraoperative opioid use, mean intraoperative BIS, and intraoperative regional block. Eleven variables were retained after backward likelihood-ratio selection, with additional intraoperative opioid use removed; the apparent AUC in the development sample was 0.835. When univariable screening and backward selection were fully embedded within 1,000 bootstrap resamples, the optimism-corrected AUC for the complete algorithmic pathway was 0.793, and the median number of predictors ultimately retained was 10 [interquartile range (IQR), 9–11]. Selection frequencies exceeded 90% for age, baseline preoperative anxiety, mean intraoperative BIS, at-risk temperament, intraoperative regional block, and mean inspired sevoflurane concentration; the selection frequency for sedative premedication was 71.7% (Table 5).

**Table 5:** Original candidate-variable screening, backward selection, and bootstrap selection stability.

Candidate predictor	Coding	Univariable odds ratio (OR), 95% confidence interval (CI)	Likelihood-ratio (LR) *P* value	Entered at *P* < 0.10	Retained after backward selection	Bootstrap selection frequency
Age	Per 1 year	0.729 (0.633–0.840)	<0.001	Yes	Yes	95.9%
Male sex	Male = 1	1.182 (0.666–2.098)	0.566	No	No	10.6%
Body mass index (BMI)	Per 1 kg/m^2^	0.950 (0.858–1.053)	0.327	No	No	10.7%
American Society of Anesthesiologists (ASA) physical status II	II = 1	1.273 (0.659–2.456)	0.476	No	No	12.0%
Bilateral hernia	Bilateral = 1	1.456 (0.862–2.462)	0.162	No	No	4.1%
Baseline preoperative anxiety	Yes = 1	3.141 (1.880–5.249)	<0.001	Yes	Yes	91.8%
Sedative premedication	Yes = 1	0.475 (0.276–0.819)	0.006	Yes	Yes	71.7%
Actual fasting duration	Per 1 h	1.156 (1.031–1.296)	0.012	Yes	Yes	73.0%
Clear-fluid fasting duration	Per 1 h	1.215 (1.038–1.420)	0.014	Yes	Yes	50.8%
Parental presence	Yes = 1	0.505 (0.303–0.843)	0.009	Yes	Yes	67.3%
At-risk temperament	Difficult/slow-to-warm-up = 1	2.597 (1.561–4.319)	<0.001	Yes	Yes	92.6%
Mean inspired sevoflurane concentration	Per 1%	2.550 (1.451–4.478)	<0.001	Yes	Yes	90.5%
Total sufentanil dose	Per 1 μg	0.843 (0.743–0.956)	0.006	Yes	Yes	67.6%
Additional intraoperative opioid	Yes = 1	0.451 (0.226–0.897)	0.017	Yes	No	46.5%
Mean intraoperative bispectral index (BIS)	Per 1 point	0.915 (0.872–0.960)	<0.001	Yes	Yes	96.4%
Intraoperative regional block	Yes = 1	0.382 (0.214–0.682)	<0.001	Yes	Yes	91.3%

Univariable *P* values are from two-sided likelihood-ratio tests. Each bootstrap sample began with all 16 candidate predictors and repeated univariable screening and backward likelihood-ratio selection; selection frequency is the proportion of final models in which a variable was retained across 1,000 resamples. The apparent AUC of the original selected model in the development sample was 0.835, and the nested bootstrap-corrected AUC for the complete algorithmic pathway was 0.793; the median number of predictors retained was 10 (IQR, 9–11).

BMI, body mass index; ASA, American Society of Anesthesiologists; BIS, bispectral index; OR, odds ratio; CI, confidence interval; LR, likelihood ratio; AUC, area under the receiver operating characteristic curve; IQR, interquartile range; bootstrap, bootstrap resampling.

Across the exploratory subgroups, AUCs for the fixed four-variable model ranged from 0.646 to 0.840. The *P* values for between-subgroup differences in AUC by age, sex, BMI stratum, ASA physical status, and hernia type were 0.146, 0.258, 0.218, 0.490, and 0.495, respectively. Some subgroups had few events, and no multiplicity adjustment was applied; these results describe discrimination across clinical strata. In Firth penalized-likelihood logistic regression, the directions of association for age, baseline preoperative anxiety, sedative premedication, and intraoperative regional block were consistent with those in the primary model (Supplementary Tables 1, 2).

## 4 Discussion

After reviewing the original screening and backward-selection results, we developed a *post hoc* four-variable exploratory model with the prediction time set immediately before planned tracheal extubation. The fixed model estimates the conditional risk of PAED-defined EA after completion of the specified preoperative and intraoperative management. The apparent AUC was 0.768, the optimism-corrected AUC was 0.755, and the optimism-corrected calibration slope was 0.943, indicating moderate discrimination and acceptable internal calibration in the development cohort. The original algorithmic selection pathway retained 11 variables in the development sample, whereas the median number retained in bootstrap samples was 10 (IQR, 9–11), with differing selection frequencies across variables. The apparent EPV of 23.3 describes the parameter burden of the final fixed model; the original data-driven selection and *post hoc* simplification may nevertheless introduce selection-related optimism (Tables 4, 5).

The incidence of PAED-defined EA in this cohort was 32.4%, within the range reported in recent studies of pediatric surgical patients (22, 23). Increasing age was associated with lower odds of EA, suggesting that younger children may be more susceptible to behavioral disturbance during emergence. Cognitive integration, verbal expression, adaptation to unfamiliar environments, and self-regulation remain immature in young children, who may therefore be more sensitive to preoperative separation, mask application, venipuncture, and stimulation in the PACU. A systematic review likewise identified younger age as an important correlate of pediatric emergence delirium (23).

Baseline preoperative anxiety was associated with higher odds of EA. Preoperative anxiety may manifest as difficulty separating from parents, avoidance of medical contact, and anticipatory concern about pain or surgery; it may heighten perioperative stress and impair cooperation during anesthesia induction (24). Nonpharmacologic interventions such as distraction can reduce preoperative anxiety in children (25). In this study, baseline anxiety was defined using the mYPAS before sedative premedication, thereby ensuring that measurement preceded drug administration and reducing the influence of medication on baseline behavioral assessment.

Intranasal dexmedetomidine, oral midazolam, and intravenous midazolam were combined as “any sedative premedication.” The mYPAS was completed before medication, establishing the temporal sequence between baseline anxiety and sedative premedication; baseline anxiety may also have influenced selection of the sedative regimen. Previous studies have compared parental presence, midazolam, dexmedetomidine, and other sedative strategies with respect to preoperative anxiety, quality of sedation, and emergence behavior (26–30). The anxiety and sedative coefficients in the model should be interpreted as conditional predictive associations after adjustment for the other predictors. Because the prediction time followed implementation of both the sedative regimen and regional block, the model estimates the conditional risk of PAED-defined EA after the specified perioperative management. Treatment effects of individual sedative regimens require prospective comparative studies with prespecified protocols.

Univariable analyses showed that the EA group had a higher mean inspired sevoflurane concentration, a lower total sufentanil dose, a lower frequency of additional intraoperative opioid administration, and lower minimum and mean BIS values. Associations of volatile anesthetic exposure, anesthetic depth, intraoperative sedation, and analgesic strategies with pediatric EA or emergence delirium are jointly influenced by age, surgical procedure, and speed of emergence (31–35). These variables had differing selection frequencies in the original data-driven pathway but were not all included in the *post hoc* simplified four-variable model. Their incremental predictive value and stability across centers should be reassessed in external cohorts.

Intraoperative regional block was associated with lower odds of EA. Transversus abdominis plane and rectus sheath blocks can attenuate nociceptive input from abdominal-wall incisions and are commonly used for analgesia in pediatric lower-abdominal or inguinal surgery (12, 13, 36). This variable reflects the perioperative management pathway at our center, and its coefficient represents a conditional predictive association. The independent treatment effect of regional block on EA may remain confounded by indication, anesthesiologist preference, surgical extent, and other analgesic measures.

Setting the prediction time immediately before planned tracheal extubation and before the first PAED assessment ensured that all model variables were available when risk was estimated, while the outcome had not yet been measured. PACU pain scores, coughing after extubation, and other recovery-phase process measures occur concurrently with or after EA and are not suitable candidate predictors at this prediction time. The PAED scale was originally developed to assess emergence delirium (16, 17); behavioral manifestations of pain, agitation, and delirium may overlap, and pediatric pain scales also rely on observable behavior (5, 18). The outcome in this study should therefore be interpreted as a PAED-defined behavioral phenotype during recovery.

The fixed four-variable model had an apparent AUC of 0.768 and an optimism-corrected AUC of 0.755, with an optimism-corrected calibration slope of 0.943 and intercept of −0.028. These findings indicate moderate discrimination and generally acceptable internal calibration in the development sample. The unrounded probability cutoff maximizing Youden J was 0.348 and was reported as the exploratory classification point of 0.35 to two decimal places to summarize classification performance in this cohort. Sensitivity, specificity, and predictive values are subject to sampling uncertainty, and predictive values depend on the incidence of EA in this cohort. Transportability should be assessed in an independent external cohort at the same prediction time, including recalibration and evaluation of net benefit using decision-curve analysis (7–10).

In exploratory subgroup analyses, AUCs ranged from 0.646 to 0.840, and no statistically significant between-subgroup differences in AUC were observed. Some subgroups contained few events, and no multiplicity adjustment was applied. The directions of association in the Firth penalized-likelihood analysis were consistent with those in the primary model, providing supplementary evidence regarding the directional robustness of the fixed-model coefficients. Future studies should prespecify subgroup comparisons in independent multicenter cohorts and report discrimination, calibration, and clinical net benefit together.

The four-variable model was simplified primarily according to availability at the prediction time, nonredundancy across variable domains, completeness of documentation, and parsimony of presentation. Because this combination was formed after the original results had been examined, it represents *post hoc* modeling; its value lies in providing a clearly structured candidate model for independent validation. Sedative premedication and intraoperative regional block were determined by clinical decisions, and their estimates may be influenced by indication, clinician preference, and local care pathways. Future work should separate external model validation from evaluation of intervention effects. The former should assess discrimination, calibration, and clinical net benefit at the same prediction time and update the model where necessary; the latter requires prospective comparative or randomized studies using prespecified treatment strategies.

This study has several limitations. First, the four-predictor combination was formed *post hoc* after the original analytical results had been reviewed. Conditional bootstrap validation of the fixed model assessed fitting optimism given the specified four-variable model, whereas nested bootstrap validation of the original algorithmic pathway assessed the repeatable selection steps; the *post hoc* clinical simplification nevertheless remains a development-stage judgment. Second, this was a single-center retrospective study, and transportability to other institutions, anesthetic pathways, and populations with different incidences of EA requires independent evaluation. Third, 28 clinically eligible records were excluded from complete-case analysis because the PAED outcome or at least one candidate predictor was missing, and missingness-related selection bias remains possible. Fourth, the prediction time followed completion of decisions regarding sedative premedication and intraoperative regional block; the model therefore estimates conditional risk under the specified management, and these two management variables may be subject to residual confounding by indication, clinician preference, and center-specific processes. Fifth, different sedative drugs and routes were combined into a single binary variable, and drug heterogeneity was not modeled separately. Sixth, dichotomizing the mYPAS at >30 may have discarded continuous information. Seventh, children aged 1–12 years were assessed using age-appropriate questionnaires from the Carey temperament series, and differences among questionnaire versions may have introduced measurement heterogeneity. Eighth, the PAED scale was originally developed to assess emergence delirium, and the phenotypes of pain, agitation, and delirium may overlap. Ninth, PAED and mYPAS ratings depend on observer judgment, and reportable interrater reliability data were unavailable in this study.

## 5 Conclusions

The *post hoc* four-predictor nomogram developed in this study used immediately before planned tracheal extubation and before the first PAED assessment as the prediction time. After conditional bootstrap internal validation, the model showed moderate discrimination and acceptable calibration in the development cohort and provides an exploratory estimate of PAED-defined EA risk after completion of the specified perioperative management. Independent multicenter external validation, model updating where necessary, and prospective evaluation of clinical impact are required before clinical implementation.

## Data Availability

The original contributions presented in the study are included in the article/Supplementary Material, further inquiries can be directed to the corresponding author.
