# Supplementary material for: A nomogram for predicting emergence agitation after laparoscopic high ligation of the hernia sac in children
Source: Front Pediatr. 2026 Aug 5;14:1847049. doi: 10.3389/fped.2026.1847049 (PMC13505351; doi:10.3389/fped.2026.1847049)
Supplement: Supplementary file 1 [file Table1.docx]

**Supplementary Material**

**Supplementary Table 1. Exploratory subgroup discrimination of the post hoc four-predictor model**

| Subgroup variable | Level | Number of children | Emergence agitation, *n* (%) | Area under the receiver operating characteristic curve (AUC), 95% confidence interval (CI) | *P* value for between-subgroup AUC difference |
| --- | --- | --- | --- | --- | --- |
| Age | <4 years | 150 | 63 (42.0) | 0.695 (0.610–0.775) | 0.146 |
| Age | ≥4 years | 137 | 30 (21.9) | 0.788 (0.688–0.880) | 0.146 |
| Sex | Female | 74 | 22 (29.7) | 0.840 (0.728–0.934) | 0.258 |
| Sex | Male | 213 | 71 (33.3) | 0.739 (0.668–0.802) | 0.258 |
| Body mass index (BMI), cutoff 18.22 kg/m² | <Cohort 85th percentile | 244 | 80 (32.8) | 0.785 (0.721–0.845) | 0.218 |
| BMI, cutoff 18.22 kg/m² | ≥Cohort 85th percentile | 43 | 13 (30.2) | 0.646 (0.474–0.818) | 0.218 |
| American Society of Anesthesiologists (ASA) physical status | ASA I | 241 | 76 (31.5) | 0.761 (0.695–0.823) | 0.490 |
| ASA physical status | ASA II | 46 | 17 (37.0) | 0.824 (0.684–0.933) | 0.490 |
| Hernia type | Unilateral | 198 | 59 (29.8) | 0.748 (0.667–0.820) | 0.495 |
| Hernia type | Bilateral | 89 | 34 (38.2) | 0.791 (0.690–0.884) | 0.495 |

**Note:** The 85th percentile of BMI was 18.22 kg/m². The 95% CIs for subgroup AUCs and *P* values for between-subgroup AUC differences were estimated using 2,000 outcome-stratified bootstrap resamples. *P* values are two-sided, with no adjustment for multiplicity. *n*, number of children; EA, emergence agitation; BMI, body mass index; ASA, American Society of Anesthesiologists; AUC, area under the receiver operating characteristic curve; CI, confidence interval.

**Supplementary Table 2. Sensitivity analysis using Firth penalized-likelihood logistic regression**

| Variable | Firth odds ratio (OR) | 95% confidence interval (CI) | *P* value |
| --- | --- | --- | --- |
| Age (per 1-year increase) | 0.728 | 0.623–0.841 | <0.001 |
| Baseline preoperative anxiety | 2.589 | 1.511–4.483 | <0.001 |
| Sedative premedication | 0.453 | 0.248–0.808 | 0.007 |
| Intraoperative regional block | 0.382 | 0.203–0.694 | 0.001 |

**Note:** The same four post hoc selected predictors as in Table 3 were used. Firth OR denotes the odds ratio obtained from Firth penalized-likelihood logistic regression; 95% CIs are profile penalized-likelihood confidence intervals, and *P* values are from two-sided penalized likelihood-ratio tests. OR, odds ratio; CI, confidence interval.
